# Alterations in reversible protein histidine phosphorylation as intracellular signals in cardiovascular disease

**DOI:** 10.3389/fphar.2015.00173

**Published:** 2015-08-21

**Authors:** Thomas Wieland, Paul V. Attwood

**Affiliations:** ^1^Institute for Experimental and Clinical Pharmacology and Toxicology, Mannheim Medical Faculty, Heidelberg University, Mannheim, Germany; ^2^School of Chemistry and Biochemistry, The University of Western Australia, Crawley, Australia

**Keywords:** protein histidine phosphorylation, nucleoside diphosphate kinase B, phosphohistidine phosphatase, heterotrimeric G-proteins, caveolae, SK4 channel, heart failure, atherosclerosis

## Abstract

Reversible phosphorylation of amino acid side chains in proteins is a frequently used mechanism in cellular signal transduction and alterations of such phosphorylation patterns are very common in cardiovascular diseases. They reflect changes in the activities of the protein kinases and phosphatases involving signaling pathways. Phosphorylation of serine, threonine, and tyrosine residues has been extensively investigated in vertebrates, whereas reversible histidine phosphorylation, a well-known regulatory signal in lower organisms, has been largely neglected as it has been generally assumed that histidine phosphorylation is of minor importance in vertebrates. More recently, it has become evident that the nucleoside diphosphate kinase isoform B (NDPK-B), an ubiquitously expressed enzyme involved in nucleotide metabolism, and a highly specific phosphohistidine phosphatase (PHP) form a regulatory histidine protein kinase/phosphatase system in mammals. At least three well defined substrates of NDPK-B are known: The β-subunit of heterotrimeric G-proteins (Gβ), the intermediate conductance potassium channel SK4 and the Ca^2+^ conducting TRP channel family member, TRPV5. In each of these proteins the phosphorylation of a specific histidine residue regulates cellular signal transduction or channel activity. This article will therefore summarize our current knowledge on protein histidine phosphorylation and highlight its relevance for cardiovascular physiology and pathophysiology.

## Histidine Phosphorylation—Histidine Kinases and Phosphohistidine Phosphatases, a General Perspective

Phosphorylation is a most ubiquitous post-translational modification that plays an essential role in the regulation of cellular function is. In higher eukaryotes the most common forms of phosphorylation of proteins involve serine/threonine or tyrosine protein kinases and counteracting phosphatases. Both types of enzymes can be regulated by cellular signaling events.

In prokaryotes, but also in fungi and plants, phosphorylation of proteins on histidine residues is very common. The most widespread occurrence of histidine phosphorylation is in the two-component histidine kinase system. The histidine kinase is a membrane-bound sensor that on perceiving an extracellular signal undergoes autophosphorylation on a histidine residue. In the simplest two-component histidine kinase systems, this phosphoryl group is then transferred directly to an aspartate residue in a response regulator protein that catalyzes the reaction. The response regulator is often a transcription factor that is activated on phosphorylation to enhance expression of genes that provide the cellular response to the external stimulus (Attwood, 2013).

**FIGURE 1:**
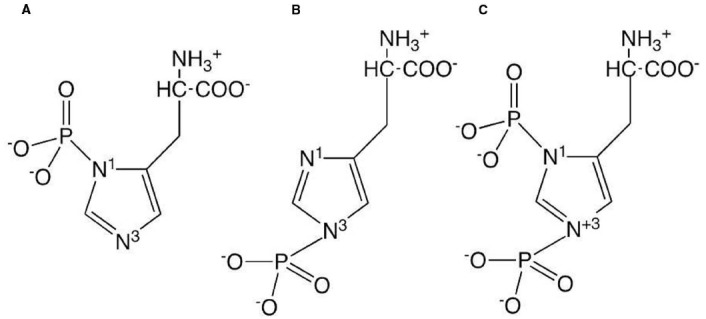
**Structures of the three forms of phosphohistidine: (A) 1-phosphohistidine; (B) 3-phosphohistidine; (C) 1,3-diphosphohistidine**.

Phosphohistidine has a number of attributes that make it different from the phosphoester phosphoamino acids. The phosphoryl group is bonded to the imidazole ring of the amino acid via a phosphoramidate bond. Since the imidazole ring contains two nitrogens, there are three forms of phosphohistidine, 1- or 3-phosphohistidine or 1,3-diphosphohistidine (see Figure 1). The two forms of monophosphohistidine are both known to occur in cellular proteins (Walinder, 1969b; Chen et al., 1977; Fujitaki et al., 1981), however the diphospho form has only been reported in chemically phosphorylated proteins. Unlike the phosphoester phosphoamino acids, all forms of phosphohistidine are unstable in acidic conditions, which has made the discovery and analysis of this type of phosphorylation technically challenging and the identification of novel proteins carrying a stable phosphohistidine are rare events (Lott et al., 2006). Nevertheless, by the use of special settings in tandem mass spectrometry (Kleinnijenhuis et al., 2007) and the description of anti-phosphohistidine antibodies (Kee and Muir, 2012; Kee et al., 2013), the detection of more proteins specifically phosphorylated on histidine residues appears more likely in the future.

In prokaryotes it is now recognized that that both serine/threonine and tyrosine kinases, as well as their cognate phosphatases also occur (for reviews, see Bakal and Davies, 2000; Pereira et al., 2011; Chao et al., 2014). Although no strong evidence for the existence of two-component histidine kinase systems in higher eukaryotes has been presented sequence databases indicate the existence of proteins in higher eukaryotes that are analogous to parts of these systems (Attwood, 2013).

It has been known for more than 50 years that protein histidine phosphorylation does occur in mammalian cells (Boyer et al., 1962; Deluca et al., 1963; Peter and Boyer, 1963). Such phosphorylation was reported in a variety of tissues: liver (Smith et al., 1973; Hegde and Das, 1987; Motojima and Goto, 1994; Noiman and Shaul, 1995); brain, lung and kidney (Noiman and Shaul, 1995); platelets (Crovello et al., 1995); trachea epithelium (Muimo et al., 2000); muscle (Rose et al., 1975). Many of these proteins are enzymes that autophosphorylate an active site histidine residue to form a kinetically competent phosphoenzyme intermediate that subsequently transfers the phosphoryl group to a substrate, e.g., phosphoglycerate mutase (Rose et al., 1975), pyruvate phosphate dikinase (Spronk et al., 1976), and nucleoside diphosphate kinase (NDPK; Walinder, 1969a). Thus many of the proteins that contain phosphohistidine are of this type and are not substrates of separate protein histidine kinases.

If histidine phosphorylation is an important post-translational modification in mammalian cell signaling, one would expect there to be phosphohistidine phosphatases. Matthews showed that some of the phosphoserine/phosphothreonine phosphatases, PP1, PP2A, and PP2C (Kim et al., 1993) are also phosphohistidine phosphatases, which utilize phosphohistidine-containing histone H4 at least as well as their phosphoserine-containing phosphoprotein substrates. These phosphatases are widely expressed in plant tissue, as well as mammalian tissue (Wong et al., 1993; Matthews and MacKintosh, 1995). More recently, a specific mammalian phosphohistidine phosphatase (PHP) has been discovered (Ek et al., 2002; Klumpp et al., 2002) and crystallized (Busam et al., 2006). This enzyme occurs in many mammalian tissues (Klumpp et al., 2002; Zhang et al., 2009). It is most highly expressed in mouse brain and in human epidermal cells, but also highly expressed in mouse and human heart and skeletal muscle (Zhang et al., 2009). It dephosphorylates of all forms of phosphohistidine (Attwood et al., 2010).

Although repeatedly described as enzymatic activity (Smith et al., 1973, 1974; Chen et al., 1974, 1977; Wei and Matthews, 1991; Besant and Attwood, 2000; Tan et al., 2004) mammalian histidine kinases remained largely unidentified and are only partially characterized so far.

**FIGURE 2:**
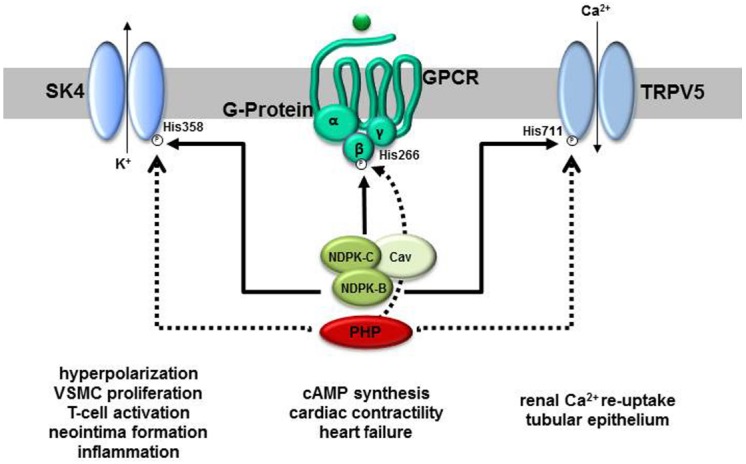
**Molecular targets of NDPK-B and PHP and their proposed functions in physiology and pathophysiology.** Three proteins, the cation channels SK4 and TRPV5 as well as the β-subunit of heterotrimeric G-proteins are substrates for NDPK-B-mediated phosphorylation on defined histidine residues (His). All three phosphohistidines are also substrates to dephosphorylation by PHP. Whereas the phosphorylation of classically regulates the open-probability of the channels, the phosphorylated G-protein β-subunit takes part in a phosphorelay activating heterotrimeric G-proteins. Apparently, by a complex formation with NDPK-C and caveolins (Cav), NDPK-B additionally contributes to caveolae formation and the composition of signaling complexes, e.g., G-protein-coupled receptor (GPCR) containing complexes, at the plasma membrane. Whereas the channels’ activities are linked to the indicated physiological and pathophysiological events, evidence for a contribution of the phosphorelay to the regulation of cardiac cAMP formation and thus contractility has been provided.

One of the first phosphoproteins to be identified that contained phosphohistidine was NDPK (Walinder, 1969a; Walinder et al., 1969). Walinder et al. (1969) showed that the phosphohistidine in NDPK was an intermediate in the phosphoryl transfer reaction between NTP and NDP catalyzed by the enzyme. More recently, it was discovered that NDPK can also act as a protein histidine kinase. NDPK-A was shown to phosphorylate a histidine residue in ATP-citrate-lyase (Wagner and Vu, 1995) whilst NDPK-B phosphorylates histidine residues in the β-subunit (Gβ) of heterotrimeric G-protein βγ-dimers (Gβγ; Cuello et al., 2003), the intermediate-conductance potassium-channel SK4 (encoded by the *KCNN4* gene; Srivastava et al., 2006) and the Ca^2+^- conducting channel, TRPV5 (Cai et al., 2014) (Figure 2). As such, NDPKs are the best characterized mammalian histidine kinases, although little is known about the details of how they recognize their substrate proteins and catalyze the phosphoryl transfer reaction. Two of the protein substrates of NDPK-B, Gβ and SK4, play important roles in cardiovascular function and disease.

## Regulation of Cardiac Contractility by NDPK-B/βγ Complex Formation

The notion that the interaction of NDPK-B with Gβγ-dimers and the histidine phosphorylation of Gβ is involved in cAMP formation in cardiac myocytes and thus the regulation of cardiac contractility became already evident in the first reports describing this interaction. Stable cell clones of immortalized neonatal rat cardiac myocyte-derived H10 cells overexpressing NDPK-B showed an enhanced Gα_*s*_-dependent activation of adenylyl cyclase (AC), which was however not seen in cells overexpressing the histidine kinase deficient NDPK mutant NDPK-B-H118N (H118) or NDPK-A (Cuello et al., 2003; Hippe et al., 2003). In membranes of these H10 cells, an increase in the content and activity of NDPK-B, as well as the formation of NDPK-B/Gβγ-complexes, was detected. This gain in functional NDPK-B/Gβγ-complexes was paralleled by an increase in intermediately phosphorylated Gβ-subunits.

Adenovirus-mediated overexpression of wild-type Gβγ-dimers, but not that of Gβγ-dimers in which the phosphorylated His266 of Gβ was mutated to leucine (GβH266Lγ), in the NDPK-B-overexpressing H10 cells further increased Gβ phosphorylation and Gα_*s*_-dependent cAMP formation (Hippe et al., 2007). GβH266Lγ, like wild-type Gβγ, was integrated into heterotrimeric G-proteins in neonatal and adult rat cardiomyocytes. However, compared to wild-type Gβγ, overexpression of GβH266Lγ suppressed basal cAMP formation, the cAMP-dependent Ser16-phosphorylation of phospholamban and contractility. A similar decrease in basal cAMP production occurred when the formation of NDPK-B/Gβγ-complexes was inhibited by siRNA-mediated NDPK-B knockdown (Hippe et al., 2007). Interestingly, in contrast to intermediately phosphorylated NDPK-B on His118, Gβ phosphorylated on His266 is a substrate for PHP and thus might be regulated also by this enzyme (Mäurer et al., 2005). Based on these data it was concluded that NDPK-B/Gβγ-complexes allow for the receptor-independent activation of G-proteins by using ATP as energy source to locally form GTP from GDP with an intermediate phosphorylation of G protein subunits at His266 (Hippe et al., 2007).

The importance of the NDPK-B/Gβγ-complex formation for cardiac contractility in zebrafish embryos has been verified by morpholino-mediated knockdown. Depletion of NDPK-B or Gβ resulted in a decrease in cardiac contractility which was associated with a reduction in the expression of the other complex partner. Moreover, the protein levels of the AC-regulating Gα_*s*_- and Gα_*i*_-subunits as well as the caveolae scaffold proteins caveolin-1 and -3 were reduced (Hippe et al., 2009). These changes were accompanied by reduced cAMP levels in the heart. Interestingly, a similar reduction of G-proteins, caveolin-1 and cAMP content was evident in embryonic fibroblasts from NDPK-A/-B double-knockout-mice. Re-expression of human NDPK-B, but not of NDPK-A, rescued this phenotype (Hippe et al., 2009). As a loss of the G_*s*_-protein at the plasma membrane should not only affect basal but also β -adrenoceptor (βAR)-induced cAMP synthesis and cardiac contractility, the requirement of NDPK-B for basal and βAR-stimulated cAMP synthesis was further analyzed by comparing wild-type NDPK-B and its catalytically inactive H118 mutant in several cellular models, including rat cardiomyocytes. Both, re-expression of human wild-type and H118 induced the re-appearance of G_*s*_ and caveolin-1 at the plasma membrane and thus enhanced the βAR-induced cAMP formation of NDPK-B—depleted cells to a similar extent. In contrast, the catalytically inactive H118 was less potent and less effective in rescuing basal cAMP production (Hippe et al., 2011a). NDPK-B thus apparently regulates G_*s*_ function by two different mechanisms. The NDPK-B-dependent phosphorelay reaction specifically allows for a receptor-independent, basal Gα_*s*_ activation and cAMP synthesis (Cuello et al., 2003; Hippe et al., 2003, 2007). The complex formation of NDPK-B with heterotrimeric G_*s*_ is additionally required for the stabilization of the G_*s*_-protein content at the plasma membrane and thus contributes to β AR-induced cAMP formation by regulating the amount of the pivotal transducer G_*s*_.

## Regulation of Caveolae Formation by NDPK-B

Caveolae are flask-shaped invaginations in the plasma membrane which are highly enriched by scaffold proteins of the caveolin and cavin families. They take part in compartmentalization and organization of signal transduction processes. In cardiomyocytes, βARs, heterotrimeric G-proteins and AC isoforms reside in caveolae (Insel et al., 2005; Insel and Patel, 2009). As the depletion of NDPK-B was associated with a loss of the expression of the caveolin isoforms-1 and -3 as well as heterotrimeric G-proteins in zebrafish embryos (Hippe et al., 2009), an interaction of NDPK-B with caveolins and thus, involvement in caveolae formation appeared likely. Similarly, in embryonic fibroblasts from the respective knockout-mice, the membrane content of caveolin-1 and NDPK-B was found to be mutually dependent on one another and a co-immunoprecipitation of caveolin-1 and NDPK-B corroborated the direct association of the two proteins (Hippe et al., 2011b). Ultrastructural analysis revealed a reduction of surface caveolae in NDPK-B-deficient cells which was associated with a decrease in the plasma membrane bound caveolin-1. In accordance with this mutual dependence between NDPK-B and caveolin, a decrease in the plasma membrane content of NDPK-B was observed in caveolin-1-deficient cells (Hippe et al., 2011b). As these alterations could be rescued by re-expression of either NDPK-B or caveolin-1 the data indicate a disturbed transport of caveolin-1- and NDPK-B-containing protein complexes from intracellular membrane compartments to the plasma membrane if one of the components is missing. Indeed, NDPK-B has been identified as part of the coat-protein-complex-II (COPII) required for vesicle transport from the endoplasmic reticulum to the Golgi apparatus (Kapetanovich et al., 2005). NDPK-B promoted the assembly of both the Sec23/24p and Sec13/31p constituents of the mammalian COPII machinery and thereby likely facilitated COPII assembly in living cells. Therefore, the authors suggested that NDPK-B is part of a scaffold-protein-complex along which ER exit sites are organized.

Caveolin-1 is also critical to other signaling cascades, e.g., the activation of the vascular endothelial growth factor (VEGF)-VEGF receptor type 2 (VEGFR-2)-cascade in endothelial cells (Sonveaux et al., 2004; Chidlow et al., 2009). Therefore, a recent report demonstrating that NDPK-B is required for VEGF-induced angiogenesis and contributes to the correct localization of VEGFR-2 and VE-cadherin at the endothelial adherens junctions (Feng et al., 2014) is interesting with regard to the importance of the NDPK-B/caveolin-1 interaction. Depletion of NDPK-B in zebrafish embryos and in cultured human endothelial cells caused malformations specifically in vessels formed by angiogenesis and impaired VEGF-induced sprouting. In accordance, NDPK-B deficient mice displayed reduced angiogenic reponses in two models of pathological vessel remodeling. Indeed, a recent abstract (Gross et al., 2015) reports that NDPK-B depletion in cultured human endothelial cells strongly reduced caveolin-1 and caveolae content at the plasma membrane and thereby impaired the VEGF-induced opening of adherence junctions. Taken together, the data indicate that NDPK-B might be required in for the functionality of many processes requiring a localization of protein complexes in caveolae and is therefore of importance for cardiovascular diseases.

## Alteration of Subcellular NDPK Localization and Function in Heart Failure

Heart failure (HF) induces complex remodeling processes in cardiomyocytes and changes in G-protein-signaling are a hallmark in this remodeling process. Chronic sympathetic stimulation results in a desensitization of βARs including reduced expression of β_1_ARs and up-regulation of inhibitory G-protein-coupled receptor-kinases (GRKs; Brinks and Koch, 2010; Santulli and Iaccarino, 2013). Concomitantly, the expression and activity of the inhibitory G_*i*_-proteins is increased by about 30% in end-stage HF (Post et al., 1999; El-Armouche et al., 2003). This is apparently associated with shift from a prevalence of Gα_*s*_-mediated AC-stimulation to Gα_*i*_-mediated inhibition (He et al., 2005). Together with the enhancement of protein phosphatase activity in HF, these alterations in cAMP signaling result in reduced phosphorylation of key cardiac Ca^2+^-handling proteins which are well established contributors to the reduced ventricular contractility that is characteristic of the disease (Schmidt et al., 1999).

The plasma membrane content of NDPK-A, -B, and -C are increased in patients with end-stage HF (Lutz et al., 2001, 2004). Chronic activation of βARs increases the association of these NDPKs to the sarcolemmal cardiomyocyte membrane whereas β-blocker treatment of HF patients obviously reduces the presence of NDPK at the plasma membrane (Lutz et al., 2003). It was speculated (Lutz et al., 2001), that the increase sarcolemmal NDPK content contributes to the prevalence of Gα_*i*_ protein signaling and thus the well-known reduction in cAMP formation in HF. A recent abstract (Abu-Taha et al., 2013) indicates that the expression of NDPK-C in cardiomyocytes is of importance for this phenomenon. NDPK-C exhibits enzymatic activity and is able to form heterohexamers with NDPK-A and NDPK-B (Gilles et al., 1991; Erent et al., 2001). It shares 72% homology with NDPK-A and NDPK-B, but has an additional hydrophobic N-terminal domain, which can serve as a membrane anchor (Morera et al., 1995; Webb et al., 1995). It is generally less abundantly expressed than the major isoforms NDPK-A and NDPK-B (Erent et al., 2001), but it is highly enriched at the cardiac plasma membrane of patients with end-stage HF and its content in these preparations reached the levels of NDPK-A and NDPK-B (Lutz et al., 2004; Abu-Taha et al., 2013). The new data revealed that it is the only isoform out of NDPK-A, -B, and -C, the expression of which is up-regulated in human HF as well as in animal and cellular models of chronic adrenergic stimulation (Abu-Taha et al., 2013). It directly interacts with heterotrimeric G-proteins and preferentially associates with cardiac Gα_*i*_ in human HF. Apparently, NDPK-C targets NDPK-heterooligomers and thus also NDPK-B to the plasma membrane and mediates the interaction with G-proteins. Future research should address whether NDPK-C is also involved in the histidine kinase activity, phosphotransfer reactions as well as the interaction with caveolins and caveolae formation.

## NDPK-B-Mediated Activation of SK4 Channels in the Vasculature is Required for Neointima Formation

Three types of Ca^2+^-activated potassium-channels, large (BK), intermediate (SK4), and small (SK3) conductance Ca^2+^-activated K^+^-channels are expressed in the vasculature (Wei et al., 2005; Feletou, 2009; Tharp and Bowles, 2009). BK-channels are preferentially found in vascular smooth muscle cells (VSMC). SK-channels are primarily expressed in the endothelium contributing to the control of vascular tone and blood pressure (Si et al., 2006; Kohler and Ruth, 2010). Unlike other cell types, VSMC are not terminally differentiated and respond to physiological as well as pathophysiological stimuli with alterations in their gene expression profile. During vasculoproliferative diseases, such as atherosclerosis and restenosis, VSMC cells undergo a phenotypic modulation characterized by suppression of contractile genes, increased proliferation, and migration. Interestingly, SK4 is one of those genes the expression of which is up-regulated during the phenotypic change from the contractile to the synthetic phenotype of VSMC and SK4-channels were functionally detected in proliferating VSMC (Toyama et al., 2008). In ApoE^–/–^-mice, a genetic model of atherosclerosis, the expression of SK4-channels was additionally increased in macrophages and T-lymphocytes that infiltrated the atherosclerotic lesions. In line with these findings, application of the selective SK4-inhibitor TRAM-34 reduced the development of atherosclerosis in these mice (Toyama et al., 2008). Also neointimal hyperplasia and stenosis after vascular injury is sensitive to TRAM-34 treatment (Kohler et al., 2003; Tharp et al., 2008) which data further suggest that the activation of SK4-channels is required for VSMC proliferation.

The activity of SK4-channels is regulated by the intracellular Ca^2+^ concentration. Ca^2+^ binds sites to calmodulin that is constitutively associated with the channel C-terminus, thereby increasing channel open-probability (Xia et al., 1998; Adelman et al., 2012). As mentioned before, NDPK-B is able to phosphorylate His358 in SK4. Although it is mechanistically not clear how this phosphorylation results in the activation of the channel, its open-probability is enhanced. In addition to the phosphorylation, the presence of phosphatidylinositol 3-phosphate [PI(3)P] is required for full activation of the channel, although the activity of NDPK-B is not dependent on the phospholipid nor does it bind to PI(3)P (Benagiano et al., 2003). The counteracting histidine phosphatase PHP also interacts with the channel and is apparently able to form a local de-/phosphorylation teeter-totter with NDPK-B (Srivastava et al., 2006, 2008; Wieland et al., 2010). Therefore, the phosphorylation and dephosphorylation of His358, the local lipid composition where the channel is inserted in the membrane as well as the Ca^2+^-concentration in vicinity to the channel are together fine-tuning its activity.

Recent evidence suggest that the activation of SK4 by NDPK-B in VSMC is required for neointima formation (Zhou et al., 2015) Using a mouse model of vessel remodeling, i.e., a guide-wire caused injury of one the carotid arteries; it was shown that NDPK-B-deficient mice were similarly protected from neointima formation in injured arteries as SK4-deficient mice. Patch clamp analysis in freshly isolated VSMC from the injured and non-injured vessel demonstrated the expression of SK4 only in VSMC from the in neointima of the injured vessel but not in VSMC of the quiescent, healthy vasculature. The current measurement (I_*SK*4_) indicated a constitutive activation of the SK4-channel in proliferating VSMCs of the wild-type but not of the NDPK-B-deficient mice. As this activation was completely inhibited by PHP, the phosphorylation of His358 in the *de novo* expressed SK4-channels by the constitutively expressed NDPK-B is pivotal to VSMC proliferation. A potential underlying mechanism is the driving of Ca^2+^-influx. In proliferating VSMC, TRPC channels and T-type Ca^2+^-channels are the main channels responsible for Ca^2+^-influx (House et al., 2008). In contrast to BK-channels, which are voltage-dependent, the voltage-independent SK4-channels can maintain channel opening even at strong negative membrane potentials. An increase in I_*SK*4_ due to the constitutive activation of the channel by NDPK-B will shift the membrane potential to more negative values. Such a hyperpolarization enhances the Ca^2+^-influx through TRPC and/or T-type Ca^2+^-channels. The resulting increase in the intracellular Ca^2+^-concentration likely triggers the activation of transcription factors and the induction of mitogenic immediate early genes (Bi et al., 2013).

Small molecules that inhibit the histidine kinase activity of NDPK-B (Buxton, 2008), interfere with its interaction with SK4 or increase PHP activity might thus offer new therapeutic options for the treatment vascular diseases such as post angioplasty restenosis. They might even be effective in the treatment of atherosclerosis. Neointima thickening in atherosclerosis does not only involve proliferation and migration of VSMC, but also activation of inflammatory cells (Libby, 2002; Niessner et al., 2006). Monocytes infiltrate the plaques, differentiate into macrophages and produce oxidative stress, proteases, and cytokines (Libby, 2002). Plasmacytoid dendritic cells in plaques activate infiltrating T-lymphocytes, which in turn further stimulate macrophages (Benagiano et al., 2003; Niessner et al., 2006). The treatment of ApoE^–/–^-mice with the selective SK4-channel blocker TRAM-34 significantly reduced not only proliferation and migration of VSMC, but also inhibited the infiltration of plaques by inflammatory cells (Toyama et al., 2008), indicating that T-lymphocyte activation in mice also requires activation of SK4-channels. Interestingly, the SK4 channel activity was reduced by 50% also in T-cells of NDPK-B-depleted mice and this caused a strong inhibition of cytokine production. As SK4^–/–^-mice are protected from developing severe colitis in mouse models of inflammatory bowel disease (Di et al., 2010a) and CD4-positive T-lymphocytes from NDPKB^–/–^- and SK4^–/–^-mice show similar profiles of cytokine production (Di et al., 2010a,b), it is very likely that the constitutive activation of SK4-channels is also required for inflammatory responses. Therefore, two important pathological processes in atherosclerosis, the proliferation of VSMC as well as inflammation and oxidative stress due to T-cell activation in the plaque, are likely to be targeted by such small molecules. Although such specific inhibitors are not known of today, they might be identified by screening compound libraries for example for inhibition of NDPK activity.

## Conclusion

Although well known as an important regulatory event in many organisms, the role of protein histidine phosphorylation in mammals has long gone unrecognized. However, with the recent identification of NDPKs as protein histidine kinases, PHP as a specific counteracting phosphatase and the description of important substrates as Gβγ, SK4, and TRPV5, an understanding of the importance of NDPK-mediated protein histidine phosphorylation in the regulation of mammalian cell function is emerging, with the promise of much more to be discovered. As pointed out herein, two of these substrates Gβγ and SK4, both targeted by NDPK-B as protein histidine kinase, have important roles in the physiology and pathophysiology of the cardiovascular system. The apparent contribution of the NDPK-B/Gβγ- and the NDPK-B/SK4-interaction to HF and atherosclerosis, respectively, give raise to the assumption that interference with the histidine kinase activity of NDPK or even more specifically, the inhibition of the interaction of the NDPK-B with its target proteins by small molecule inhibitors might offer avenues for future treatments of cardiovascular diseases.

### Conflict of Interest Statement

The authors declare that the research was conducted in the absence of any commercial or financial relationships that could be construed as a potential conflict of interest.
